# Crystal structure of *cis*-2-(2-carb­oxy­cyclo­prop­yl)glycine (CCG-III) monohydrate

**DOI:** 10.1107/S2056989015011500

**Published:** 2015-06-24

**Authors:** Sergey Lindeman, Nathaniel J. Wallock, William A. Donaldson

**Affiliations:** a Department of Chemistry, Marquette University, PO Box 1881, Milwaukee, WI 53201-1881, USA

**Keywords:** crystal structure, cyclo­propane, conformationally restricted glutamate analog

## Abstract

The title compound, C_6_H_9_NO_4_·H_2_O [systematic name: (α*R*,1*R*,2*S*)-*rel*-α-amino-2-carb­oxy­cyclo­propane­acetic acid monohydrate], crystallizes with two organic mol­ecules and two water mol­ecules in the asymmetric unit. The space group is *P*2_1_ and the organic mol­ecules are enanti­omers, thus this is an example of a ‘false conglomerate’ with two mol­ecules of opposite handedness in the asymmetric unit (r.m.s. overlay fit = 0.056 Å for one mol­ecule and its inverted partner). Each mol­ecule exists as a zwitterion, with proton transfer from the amino acid carb­oxy­lic acid group to the amine group. In the crystal, the components are linked by N—H⋯O and O—H⋯O hydrogen bonds, generating (100) sheets. Conformationally restricted glutamate analogs are of inter­est due to their selective activation of different glutamate receptors, and the naturally occurring (+)-CCG-III is an inhibitor of glutamate uptake and the key geometrical parameters are discussed.

## Abstract 2

The title compound is an example of a ‘false conglomerate’ with two mol­ecules of opposite handedness in the asymmetric unit. Each mol­ecule exists as a zwitterion, with proton transfer from the amino acid carb­oxy­lic acid group to the amine group. In the crystal, the components are linked by N—H⋯O and O—H⋯O hydrogen bonds, generating (100) sheets.

## Chemical context   

**Figure 1:**
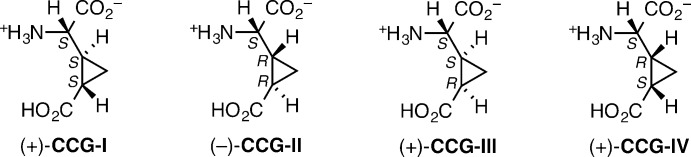
Structures of the diastereomers of 2-(2′-carb­oxy­cyclo­prop­yl)glycine.

2-(2′-Carb­oxy­cyclo­prop­yl)glycines **CCG-I**, **CCG-III** and **CCG-IV** (Fig. 1 ▸) are naturally occuring conformationally restricted analogs of glutamate isolated from *Aesculus parviflora*, *Blighia sapida* (Fowden, *et al.*, 1969 ▸), *Ephedra foeminea* (Caveney & Starratt, 1994 ▸), and *Ephedra altissima* (Starratt & Caveney, 1995 ▸). While not naturally occurring, both enanti­omers of **CCG-II** (Fig. 1 ▸) have been prepared in the laboratory (Shimamoto, *et al.*, 1991 ▸) and all of the diastereomeric CCGs are useful tools for investigating the mechanism of glutamate function. The crystal structure of the title hydrate, (±)-**CCG-III**·H_2_O, is now reported.
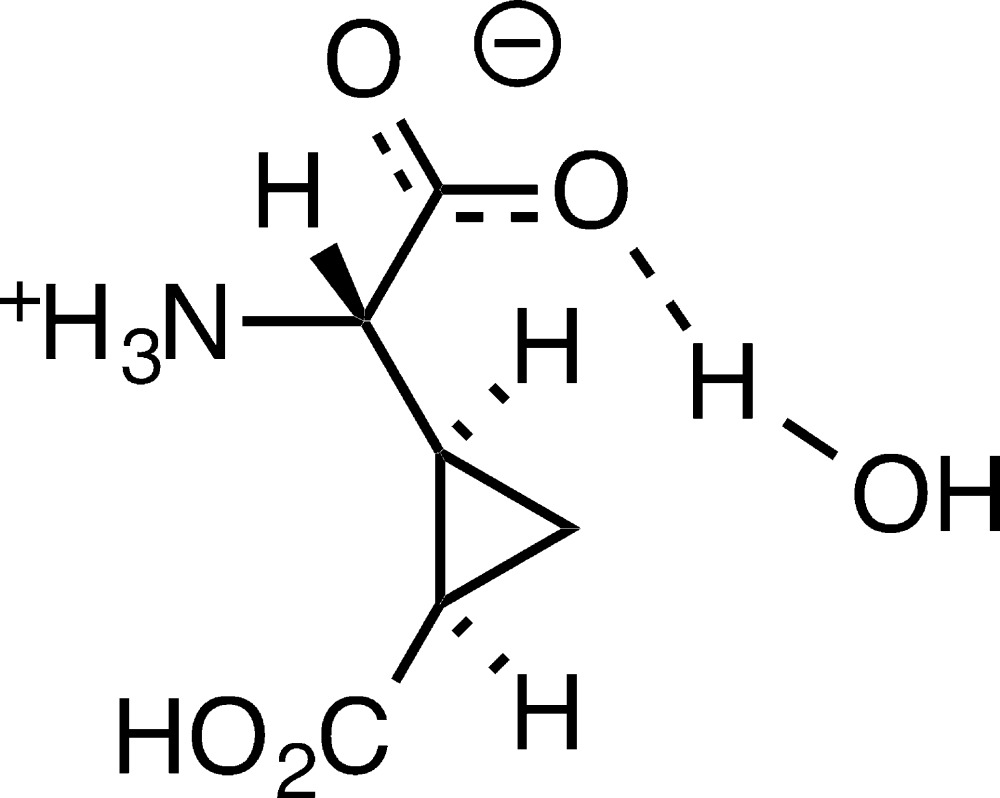


## Structural commentary   

**Figure 2:**
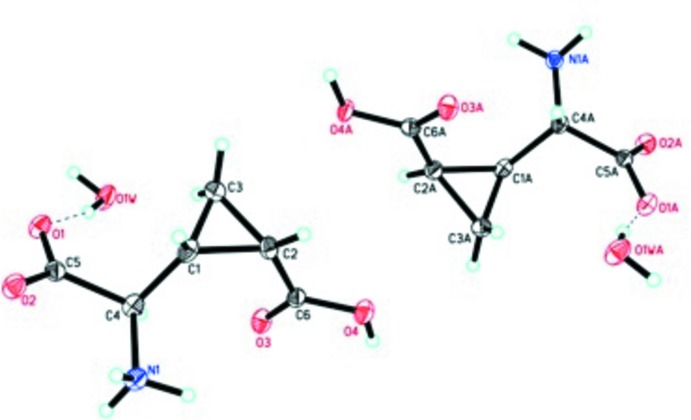
The asymmetic unit of the title compound, showing 50% displacement ellipsoids.

The racemic title compound (Fig. 2 ▸) crystallizes as a ‘false conglomerate’ with two mol­ecules of opposite handedness in the asymmetric unit. Each of mol­ecules of 2-(2′-carb­oxy­cyclo­prop­yl)glycine has a mol­ecule of water hydrogen bonded to the glycine carboxyl­ate group. It has been estimated that only 1% of organic compounds are false conglomerates (Bishop & Scudder, 2009 ▸).

The torsion angles O3—C6—C2—*X =* −4.3° and O3*A*—C6*A*—C2*A*—*X =* −11.1° (where *X* is the midpoint of the distal cyclo­propane bond) indicate that the carb­oxy­lic acid attached to the cyclpropane ring adopts a bis­ected conform­ation (Allen, 1980 ▸). The cyclo­propane C—C bonds proximal to the C2 carb­oxy­lic group are roughly equal [C1—C2 = 1.532 (3); C2—C3 = 1.512 (3); C1*A*—C2*A* = 1.520 (3); C2*A*—C3*A* = 1.516 (2) Å] and are longer than the cyclo­propane bonds distal to the C2 carb­oxy­lic acid [C1—C3 = 1.489 (2); C1*A*—C3*A* = 1.484 (2) Å]. These distances and torsion angles are consistent with other cyclo­propane carb­oxy­lic acids (Allen, 1980 ▸).

Conformationally restricted glutamic acid analogs can be classified into one of four categories, which are characterized by the distances between the nitro­gen atom of the amino group and the γ-carboxyl­ate carbon atom (*d_1_*), between the α- and γ-carboxyl­ate carbon atoms (*d_2_*), and their sum (*d_1_* + *d_2_*). The classifications ‘folded’, ‘semi-folded’, ‘semi-extended’, and ‘extended’ are defined by (*d_1_* + *d*_2_) ≤ 7.5 Å, 7.5 Å ≤ (*d_1_* + *d*_2_) ≤ 8.0 Å, 8.0 Å ≤ (*d_1_* + *d*_2_) ≤ 8.5 Å, and (*d_1_* + *d*_2_) ≥ 8.5 Å, respectively (Pellicciari, *et al.*, 2002 ▸). The two enanti­omeric moleclules in the crystal structure evidence the following distances/sums: *d_1_*, 3.65 and 3.71 Å; *d_2_*, 4.59 and 4.59 Å; (*d_1_* + *d_2_*), 8.24 and 8.30 Å, respectively. From these values, these conformers of CCG-III can be considered to be in the ‘semi-extended’ class.

## Supra­molecular features   

**Figure 3:**
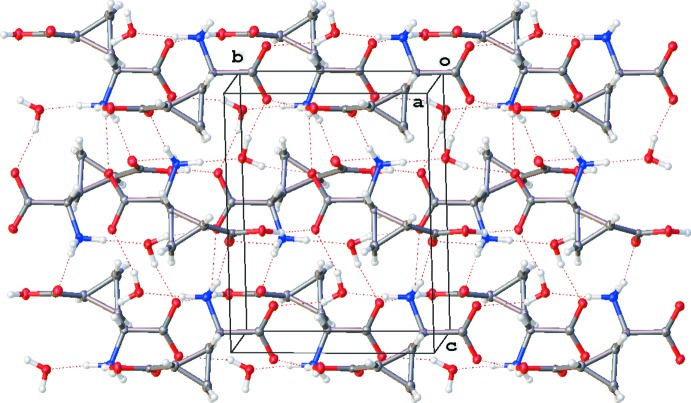
The packing for the title compound viewed approximately down [100], with hydrogen bonds shown as dashed lines.

In the crystal, the mol­ecules are linked by N—H⋯O and O—H⋯O hydrogen bonds, forming sheets parallel to (100); Table 1 ▸ and Fig. 3 ▸.

## Synthesis and crystallization   

The racemic title compound was prepared according to the literature procedure (Wallock & Donaldson, 2004 ▸). A sample for X-ray diffraction analysis was recrystallized from water.

## Refinement   

Crystal data, data collection and structure refinement details are summarized in Table 2 ▸.

## Supplementary Material

Crystal structure: contains datablock(s) I, New_Global_Publ_Block. DOI: 10.1107/S2056989015011500/hb7407sup1.cif

Structure factors: contains datablock(s) I. DOI: 10.1107/S2056989015011500/hb7407Isup2.hkl

Supporting information file. DOI: 10.1107/S2056989015011500/hb7407Isup3.cml

CCDC reference: 1406594

Additional supporting information:  crystallographic information; 3D view; checkCIF report

## Figures and Tables

**Table 1:** Hydrogen-bond geometry (, )

*D*H*A*	*D*H	H*A*	*D* *A*	*D*H*A*
N1H1*A*O3*A* ^i^	0.94(2)	2.03(2)	2.9444(18)	162.1(17)
N1H1*B*O2*A* ^ii^	0.86(2)	2.39(2)	2.9454(18)	123.1(16)
N1H1*C*O1*WA* ^i^	0.98(3)	1.83(3)	2.795(2)	167(2)
O4H4O1^iii^	0.81(3)	1.79(3)	2.5851(18)	166(3)
O1*W*H1*WA*O2*A* ^iv^	0.82(3)	2.01(3)	2.8072(19)	166(2)
O1*W*H1*WB*O1	0.86(2)	1.90(2)	2.7449(16)	169(2)
N1*A*H1*AA*O3^v^	0.90(2)	2.01(2)	2.9087(18)	173(2)
N1*A*H1*AB*O3*A* ^vi^	0.87(2)	2.38(2)	3.1151(19)	141.7(17)
N1*A*H1*AC*O1*W* ^v^	0.93(2)	1.87(2)	2.785(2)	165.6(18)
O4*A*H4*AA*O1*A* ^vii^	0.98(3)	1.60(3)	2.5672(16)	168(2)
O1*WA*H1*WC*O2^viii^	0.83(3)	2.07(3)	2.8628(19)	158(2)
O1*WA*H1*WD*O1*A*	0.81(2)	1.98(3)	2.7717(17)	166(3)

Symmetry codes: (i) 
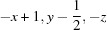
; (ii) 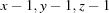; (iii) 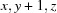; (iv) 
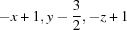
; (v) 
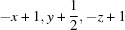
; (vi) 
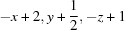
; (vii) 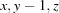; (viii) 
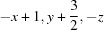
.

**Table 2:** Experimental details

Crystal data	
Chemical formula	C_6_H_9_NO_4_H_2_O
*M*_r_	177.16
Crystal system, space group	Monoclinic, *P*2_1_
Temperature (K)	100
*a*, *b*, *c* ()	8.9688(8), 8.0063(8), 10.9628(10)
()	106.015(4)
*V* (^3^)	756.65(12)
*Z*	4
Radiation type	Cu *K*
(mm^1^)	1.18
Crystal size (mm)	0.37 0.32 0.10

Data collection	
Diffractometer	Bruker APEXII CCD detector
Absorption correction	Multi-scan (*SADABS*; Bruker, 2005 ▸)
*T*_min_, *T*_max_	0.669, 0.891
No. of measured, independent and observed [*I* > 2(*I*)] reflections	6086, 2164, 2154
*R*_int_	0.018
_max_ ()	61.0
(sin /)_max_ (^1^)	0.567

Refinement	
*R*[*F* ^2^ > 2(*F* ^2^)], *wR*(*F* ^2^), *S*	0.021, 0.055, 1.06
No. of reflections	2164
No. of parameters	305
No. of restraints	1
H-atom treatment	All H-atom parameters refined
_max_, _min_ (e ^3^)	0.15, 0.16
Absolute structure	Flack (1983 ▸), 836 Friedel pairs
Absolute structure parameter	0.57(15)

Computer programs: *APEX2* and *SAINT* (Bruker, 2005 ▸), *SHELXTL* and *SHELXL97* (Sheldrick, 2008 ▸).

## supplementary crystallographic information

### Crystal data

**Table:**

C_6_H_9_NO_4_·H_2_O	*F*(000) = 376
*M**_r_* = 177.16	*D*_x_ = 1.555 Mg m^−^^3^
Monoclinic, *P*2_1_	Cu *K*α radiation, λ = 1.54178 Å
*a* = 8.9688 (8) Å	Cell parameters from 5577 reflections
*b* = 8.0063 (8) Å	θ = 4–61°
*c* = 10.9628 (10) Å	µ = 1.18 mm^−^^1^
β = 106.015 (4)°	*T* = 100 K
*V* = 756.65 (12) Å^3^	Plate, colorless
*Z* = 4	0.37 × 0.32 × 0.10 mm

### Data collection

**Table:**

Bruker APEXII CCD detector diffractometer	2164 independent reflections
Radiation source: fine-focus sealed tube	2154 reflections with *I* > 2σ(*I*)
Graphite monochromator	*R*_int_ = 0.018
ω scans	θ_max_ = 61.0°, θ_min_ = 4.2°
Absorption correction: multi-scan (*SADABS*; Bruker, 2005)	*h* = −10→9
*T*_min_ = 0.669, *T*_max_ = 0.891	*k* = −8→9
6086 measured reflections	*l* = 0→12

### Refinement

**Table:**

Refinement on *F*^2^	Secondary atom site location: difference Fourier map
Least-squares matrix: full	Hydrogen site location: difference Fourier map
*R*[*F*^2^ > 2σ(*F*^2^)] = 0.021	All H-atom parameters refined
*wR*(*F*^2^) = 0.055	*w* = 1/[σ^2^(*F*_o_^2^) + (0.0523*P*)^2^ + 0.0652*P*] where *P* = (*F*_o_^2^ + 2*F*_c_^2^)/3
*S* = 1.06	(Δ/σ)_max_ < 0.001
2164 reflections	Δρ_max_ = 0.15 e Å^−^^3^
305 parameters	Δρ_min_ = −0.16 e Å^−^^3^
1 restraint	Absolute structure: Flack (1983), 836 Friedel pairs
Primary atom site location: structure-invariant direct methods	Absolute structure parameter: 0.57 (15)

### Special details

**Table:**

Geometry. All e.s.d.'s (except the e.s.d. in the dihedral angle between two l.s. planes) are estimated using the full covariance matrix. The cell e.s.d.'s are taken into account individually in the estimation of e.s.d.'s in distances, angles and torsion angles; correlations between e.s.d.'s in cell parameters are only used when they are defined by crystal symmetry. An approximate (isotropic) treatment of cell e.s.d.'s is used for estimating e.s.d.'s involving l.s. planes.
Refinement. Refinement of *F*^2^ against ALL reflections. The weighted *R*-factor *wR* and goodness of fit *S* are based on *F*^2^, conventional *R*-factors *R* are based on *F*, with *F* set to zero for negative *F*^2^. The threshold expression of *F*^2^ > σ(*F*^2^) is used only for calculating *R*-factors(gt) *etc*. and is not relevant to the choice of reflections for refinement. *R*-factors based on *F*^2^ are statistically about twice as large as those based on *F*, and *R*- factors based on ALL data will be even larger.

### Fractional atomic coordinates and isotropic or equivalent isotropic displacement parameters (Å^2^)

**Table:**

	*x*	*y*	*z*	*U*_iso_*/*U*_eq_	
O1	0.16905 (12)	−0.14281 (15)	0.09397 (9)	0.0171 (3)	
O2	0.16680 (12)	−0.14746 (15)	−0.11118 (10)	0.0182 (3)	
O3	0.17732 (12)	0.43933 (15)	0.13921 (10)	0.0179 (3)	
O4	0.36153 (14)	0.61213 (15)	0.11160 (11)	0.0187 (3)	
N1	0.10810 (17)	0.18398 (19)	−0.14010 (12)	0.0163 (3)	
C1	0.34487 (17)	0.1610 (2)	0.03879 (15)	0.0167 (3)	
C2	0.40767 (18)	0.3278 (2)	0.10039 (15)	0.0169 (3)	
C3	0.42184 (19)	0.1692 (3)	0.17765 (16)	0.0203 (4)	
C4	0.17508 (18)	0.1174 (2)	−0.00932 (14)	0.0147 (4)	
C5	0.16579 (16)	−0.0746 (2)	−0.01143 (14)	0.0143 (4)	
C6	0.30359 (18)	0.4620 (2)	0.11880 (14)	0.0139 (4)	
H1A	0.150 (2)	0.128 (3)	−0.1987 (17)	0.020 (5)*	
H1B	0.010 (3)	0.166 (3)	−0.1641 (18)	0.029 (5)*	
H1C	0.131 (3)	0.303 (4)	−0.143 (2)	0.050 (7)*	
H4	0.297 (3)	0.681 (4)	0.115 (2)	0.044 (7)*	
H1	0.407 (2)	0.114 (2)	−0.0118 (16)	0.020 (5)*	
H2	0.499 (2)	0.364 (2)	0.0793 (15)	0.017 (4)*	
H3A	0.522 (2)	0.127 (3)	0.2107 (17)	0.020 (4)*	
H3B	0.354 (2)	0.154 (3)	0.2312 (15)	0.017 (4)*	
H4A	0.1176 (17)	0.159 (3)	0.0430 (14)	0.006 (4)*	
O1W	0.15198 (15)	−0.01307 (17)	0.32148 (12)	0.0218 (3)	
H1WA	0.152 (3)	−0.106 (3)	0.352 (2)	0.038 (7)*	
H1WB	0.149 (2)	−0.042 (3)	0.246 (2)	0.034 (6)*	
O1A	0.81679 (12)	1.13479 (15)	0.40666 (10)	0.0161 (3)	
O2A	0.86473 (13)	1.14792 (16)	0.61824 (10)	0.0175 (3)	
O3A	0.80378 (12)	0.55765 (16)	0.36274 (10)	0.0177 (3)	
O4A	0.63822 (12)	0.38208 (15)	0.41642 (10)	0.0162 (3)	
N1A	0.92671 (16)	0.8177 (2)	0.64801 (12)	0.0142 (3)	
C1A	0.66918 (17)	0.8314 (2)	0.49476 (14)	0.0144 (4)	
C2A	0.59847 (17)	0.6653 (2)	0.44103 (14)	0.0156 (3)	
C3A	0.56440 (18)	0.8249 (2)	0.36381 (16)	0.0173 (4)	
C4A	0.83802 (17)	0.8786 (2)	0.51986 (14)	0.0133 (4)	
C5A	0.84454 (16)	1.0708 (2)	0.51702 (14)	0.0129 (4)	
C6A	0.69085 (18)	0.5329 (2)	0.40323 (13)	0.0149 (4)	
H1AA	0.892 (2)	0.864 (3)	0.710 (2)	0.028 (5)*	
H1AB	1.025 (2)	0.842 (3)	0.6644 (16)	0.018 (4)*	
H1AC	0.917 (2)	0.703 (3)	0.6570 (18)	0.024 (5)*	
H4AA	0.709 (3)	0.295 (3)	0.404 (2)	0.044 (6)*	
H1AD	0.6284 (17)	0.868 (2)	0.5597 (16)	0.008 (4)*	
H2A	0.522 (2)	0.630 (2)	0.4774 (15)	0.013 (4)*	
H3AA	0.6122 (17)	0.830 (3)	0.2982 (15)	0.006 (4)*	
H3AB	0.465 (2)	0.868 (2)	0.3501 (14)	0.011 (4)*	
H4AB	0.8848 (19)	0.827 (3)	0.4588 (16)	0.016 (4)*	
O1WA	0.79114 (15)	1.00902 (16)	0.16619 (12)	0.0216 (3)	
H1WC	0.796 (3)	1.099 (3)	0.130 (2)	0.041 (7)*	
H1WD	0.804 (3)	1.030 (3)	0.241 (2)	0.041 (7)*	

### Atomic displacement parameters (Å^2^)

**Table:**

	*U*^11^	*U*^22^	*U*^33^	*U*^12^	*U*^13^	*U*^23^
O1	0.0227 (6)	0.0139 (7)	0.0160 (6)	0.0011 (5)	0.0077 (4)	0.0020 (5)
O2	0.0242 (6)	0.0144 (7)	0.0163 (6)	0.0019 (5)	0.0061 (4)	−0.0010 (5)
O3	0.0197 (6)	0.0162 (7)	0.0203 (6)	−0.0010 (5)	0.0093 (5)	−0.0007 (5)
O4	0.0202 (6)	0.0130 (7)	0.0240 (6)	−0.0006 (5)	0.0082 (5)	−0.0009 (5)
N1	0.0187 (8)	0.0155 (9)	0.0154 (7)	0.0016 (7)	0.0057 (6)	0.0006 (6)
C1	0.0181 (8)	0.0125 (9)	0.0217 (8)	0.0024 (7)	0.0090 (6)	0.0006 (8)
C2	0.0145 (7)	0.0144 (9)	0.0217 (8)	−0.0031 (7)	0.0051 (6)	0.0013 (7)
C3	0.0160 (8)	0.0160 (9)	0.0258 (9)	−0.0004 (8)	0.0006 (7)	0.0015 (8)
C4	0.0188 (8)	0.0143 (10)	0.0129 (8)	0.0016 (7)	0.0075 (7)	0.0003 (6)
C5	0.0116 (7)	0.0148 (10)	0.0164 (9)	0.0006 (7)	0.0036 (6)	−0.0009 (7)
C6	0.0180 (9)	0.0130 (9)	0.0098 (7)	−0.0023 (7)	0.0022 (6)	0.0011 (6)
O1W	0.0361 (7)	0.0127 (7)	0.0171 (6)	0.0008 (6)	0.0081 (5)	−0.0008 (5)
O1A	0.0212 (6)	0.0127 (7)	0.0160 (5)	0.0013 (5)	0.0080 (4)	0.0014 (5)
O2A	0.0230 (6)	0.0144 (6)	0.0145 (5)	0.0006 (5)	0.0039 (4)	−0.0032 (5)
O3A	0.0196 (6)	0.0160 (7)	0.0203 (6)	−0.0017 (5)	0.0099 (5)	−0.0011 (5)
O4A	0.0178 (5)	0.0092 (7)	0.0228 (6)	−0.0013 (5)	0.0074 (5)	0.0003 (5)
N1A	0.0147 (7)	0.0116 (9)	0.0170 (7)	0.0001 (6)	0.0057 (6)	−0.0005 (6)
C1A	0.0184 (8)	0.0107 (9)	0.0159 (8)	0.0015 (7)	0.0076 (6)	0.0018 (7)
C2A	0.0143 (8)	0.0151 (9)	0.0177 (7)	−0.0001 (7)	0.0050 (6)	0.0011 (7)
C3A	0.0144 (8)	0.0155 (10)	0.0215 (8)	0.0015 (7)	0.0044 (7)	0.0006 (7)
C4A	0.0154 (8)	0.0112 (10)	0.0140 (8)	0.0005 (7)	0.0052 (6)	−0.0006 (6)
C5A	0.0101 (7)	0.0131 (10)	0.0166 (9)	0.0004 (7)	0.0056 (6)	0.0011 (7)
C6A	0.0155 (8)	0.0163 (10)	0.0108 (7)	−0.0012 (7)	0.0000 (6)	0.0015 (7)
O1WA	0.0342 (7)	0.0147 (7)	0.0160 (6)	0.0014 (6)	0.0068 (5)	−0.0003 (6)

### Geometric parameters (Å, º)

**Table:**

O1—C5	1.271 (2)	O1A—C5A	1.273 (2)
O2—C5	1.2417 (19)	O2A—C5A	1.239 (2)
O3—C6	1.2270 (19)	O3A—C6A	1.2289 (19)
O4—C6	1.320 (2)	O4A—C6A	1.319 (2)
O4—H4	0.81 (3)	O4A—H4AA	0.98 (3)
N1—C4	1.492 (2)	N1A—C4A	1.492 (2)
N1—H1A	0.94 (2)	N1A—H1AA	0.90 (2)
N1—H1B	0.86 (2)	N1A—H1AB	0.87 (2)
N1—H1C	0.98 (3)	N1A—H1AC	0.93 (2)
C1—C2	1.532 (3)	C1A—C2A	1.520 (3)
C1—C3	1.489 (2)	C1A—C3A	1.484 (2)
C1—C4	1.509 (2)	C1A—C4A	1.510 (2)
C1—H1	0.963 (19)	C1A—H1AD	0.933 (17)
C2—C3	1.512 (3)	C2A—C3A	1.516 (2)
C2—C6	1.473 (2)	C2A—C6A	1.474 (3)
C2—H2	0.953 (18)	C2A—H2A	0.925 (17)
C3—H3A	0.93 (2)	C3A—H3AA	0.934 (16)
C3—H3B	0.965 (18)	C3A—H3AB	0.929 (18)
C4—C5	1.539 (2)	C4A—C5A	1.541 (2)
C4—H4A	0.932 (17)	C4A—H4AB	0.976 (19)
O1W—H1WA	0.82 (3)	O1WA—H1WC	0.83 (3)
O1W—H1WB	0.86 (2)	O1WA—H1WD	0.81 (2)
			
C6—O4—H4	108.5 (19)	C6A—O4A—H4AA	111.5 (14)
C4—N1—H1A	110.8 (12)	C4A—N1A—H1AA	111.9 (13)
C4—N1—H1B	110.0 (14)	C4A—N1A—H1AB	111.6 (12)
C4—N1—H1C	110.3 (14)	C4A—N1A—H1AC	112.4 (11)
H1A—N1—H1B	106.2 (19)	H1AA—N1A—H1AB	107.1 (18)
H1A—N1—H1C	108.4 (19)	H1AA—N1A—H1AC	105 (2)
H1B—N1—H1C	111 (2)	H1AB—N1A—H1AC	108.5 (19)
C2—C1—H1	113.3 (11)	C2A—C1A—H1AD	111.2 (10)
C3—C1—C2	60.03 (12)	C3A—C1A—C2A	60.61 (11)
C3—C1—C4	120.41 (14)	C3A—C1A—C4A	121.37 (13)
C3—C1—H1	115.3 (10)	C3A—C1A—H1AD	118.2 (9)
C4—C1—C2	124.66 (14)	C4A—C1A—C2A	125.40 (14)
C4—C1—H1	113.3 (10)	C4A—C1A—H1AD	111.5 (9)
C1—C2—H2	113.0 (11)	C1A—C2A—H2A	112.3 (11)
C3—C2—C1	58.57 (11)	C3A—C2A—C1A	58.51 (11)
C3—C2—H2	116.3 (11)	C3A—C2A—H2A	116.0 (11)
C6—C2—C1	121.77 (14)	C6A—C2A—C1A	122.11 (13)
C6—C2—C3	119.62 (14)	C6A—C2A—C3A	119.45 (14)
C6—C2—H2	115.7 (11)	C6A—C2A—H2A	116.1 (11)
C1—C3—C2	61.40 (11)	C1A—C3A—C2A	60.89 (12)
C1—C3—H3A	120.3 (11)	C1A—C3A—H3AA	116.1 (9)
C1—C3—H3B	115.1 (10)	C1A—C3A—H3AB	117.8 (10)
C2—C3—H3A	116.6 (12)	C2A—C3A—H3AA	113.7 (12)
C2—C3—H3B	118.6 (12)	C2A—C3A—H3AB	115.9 (11)
H3A—C3—H3B	114.6 (15)	H3AA—C3A—H3AB	119.0 (14)
N1—C4—C1	110.69 (13)	N1A—C4A—C1A	109.69 (13)
N1—C4—C5	109.68 (13)	N1A—C4A—C5A	109.39 (13)
N1—C4—H4A	108.6 (10)	N1A—C4A—H4AB	106.7 (11)
C1—C4—C5	106.36 (14)	C1A—C4A—C5A	106.73 (14)
C1—C4—H4A	112.3 (10)	C1A—C4A—H4AB	111.6 (11)
C5—C4—H4A	109.2 (12)	C5A—C4A—H4AB	112.7 (13)
O1—C5—C4	115.33 (14)	O1A—C5A—C4A	114.98 (14)
O2—C5—O1	126.49 (17)	O2A—C5A—O1A	126.36 (17)
O2—C5—C4	117.97 (14)	O2A—C5A—C4A	118.46 (14)
O3—C6—O4	122.97 (16)	O3A—C6A—O4A	122.89 (16)
O3—C6—C2	124.64 (16)	O3A—C6A—C2A	124.66 (17)
O4—C6—C2	112.39 (14)	O4A—C6A—C2A	112.45 (14)
H1WA—O1W—H1WB	99 (2)	H1WC—O1WA—H1WD	107 (3)
			
N1—C4—C5—O1	−157.34 (12)	N1A—C4A—C5A—O1A	159.17 (12)
N1—C4—C5—O2	27.53 (18)	N1A—C4A—C5A—O2A	−25.74 (18)
C1—C2—C6—O3	31.9 (2)	C1A—C2A—C6A—O3A	−30.4 (2)
C1—C2—C6—O4	−148.40 (15)	C1A—C2A—C6A—O4A	150.15 (14)
C1—C4—C5—O1	82.93 (15)	C1A—C4A—C5A—O1A	−82.23 (15)
C1—C4—C5—O2	−92.20 (16)	C1A—C4A—C5A—O2A	92.87 (16)
C2—C1—C4—N1	83.47 (18)	C2A—C1A—C4A—N1A	−85.57 (18)
C2—C1—C4—C5	−157.45 (15)	C2A—C1A—C4A—C5A	156.02 (14)
C3—C1—C2—C6	−107.63 (17)	C3A—C1A—C2A—C6A	107.23 (16)
C3—C1—C4—N1	156.11 (16)	C3A—C1A—C4A—N1A	−159.79 (16)
C3—C1—C4—C5	−84.81 (19)	C3A—C1A—C4A—C5A	81.8 (2)
C3—C2—C6—O3	−37.4 (2)	C3A—C2A—C6A—O3A	38.8 (2)
C3—C2—C6—O4	142.30 (14)	C3A—C2A—C6A—O4A	−140.58 (14)
C4—C1—C2—C3	108.16 (17)	C4A—C1A—C2A—C3A	−109.43 (17)
C4—C1—C2—C6	0.5 (2)	C4A—C1A—C2A—C6A	−2.2 (2)
C4—C1—C3—C2	−115.01 (18)	C4A—C1A—C3A—C2A	115.80 (19)
C6—C2—C3—C1	111.25 (16)	C6A—C2A—C3A—C1A	−111.72 (16)

### Hydrogen-bond geometry (Å, º)

**Table:**

*D*—H···*A*	*D*—H	H···*A*	*D*···*A*	*D*—H···*A*
N1—H1*A*···O3*A*^i^	0.94 (2)	2.03 (2)	2.9444 (18)	162.1 (17)
N1—H1*B*···O2*A*^ii^	0.86 (2)	2.39 (2)	2.9454 (18)	123.1 (16)
N1—H1*C*···O1*WA*^i^	0.98 (3)	1.83 (3)	2.795 (2)	167 (2)
O4—H4···O1^iii^	0.81 (3)	1.79 (3)	2.5851 (18)	166 (3)
O1*W*—H1*WA*···O2*A*^iv^	0.82 (3)	2.01 (3)	2.8072 (19)	166 (2)
O1*W*—H1*WB*···O1	0.86 (2)	1.90 (2)	2.7449 (16)	169 (2)
N1*A*—H1*AA*···O3^v^	0.90 (2)	2.01 (2)	2.9087 (18)	173 (2)
N1*A*—H1*AB*···O3*A*^vi^	0.87 (2)	2.38 (2)	3.1151 (19)	141.7 (17)
N1*A*—H1*AC*···O1*W*^v^	0.93 (2)	1.87 (2)	2.785 (2)	165.6 (18)
O4*A*—H4*AA*···O1*A*^vii^	0.98 (3)	1.60 (3)	2.5672 (16)	168 (2)
O1*WA*—H1*WC*···O2^viii^	0.83 (3)	2.07 (3)	2.8628 (19)	158 (2)
O1*WA*—H1*WD*···O1*A*	0.81 (2)	1.98 (3)	2.7717 (17)	166 (3)

Symmetry codes: (i) −*x*+1, *y*−1/2, −*z*; (ii) *x*−1, *y*−1, *z*−1; (iii) *x*, *y*+1, *z*; (iv) −*x*+1, *y*−3/2, −*z*+1; (v) −*x*+1, *y*+1/2, −*z*+1; (vi) −*x*+2, *y*+1/2, −*z*+1; (vii) *x*, *y*−1, *z*; (viii) −*x*+1, *y*+3/2, −*z*.
